# Demystifying Deep Learning Decisions in Leukemia Diagnostics Using Explainable AI

**DOI:** 10.3390/diagnostics16020212

**Published:** 2026-01-09

**Authors:** Shahd H. Altalhi, Salha M. Alzahrani

**Affiliations:** Department of Computer Science, College of Computers and Information Technology, Taif University, Taif 21944, Saudi Arabia; s44580237@students.tu.edu.sa

**Keywords:** Explainable Artificial Intelligence (XAI), Acute Lymphoblastic Leukemia (ALL), Acute Myeloid Leukemia (AML), Chronic Lymphocytic Leukemia (CLL), Chronic Myeloid Leukemia (CML)

## Abstract

**Background/Objectives**: Conventional workflows, peripheral blood smears, and bone marrow assessment supplemented by LDI-PCR, molecular cytogenetics, and array-CGH, are expert-driven in the face of biological and imaging variability. **Methods**: We propose an AI pipeline that integrates convolutional neural networks (CNNs) and transfer learning-based models with two explainable AI (XAI) approaches, LIME and Grad-Cam, to deliver both high diagnostic accuracy and transparent rationale. Seven public sources were curated into a unified benchmark (66,550 images) covering ALL, AML, CLL, CML, and healthy controls; images were standardized, ROI-cropped, and split with stratification (80/10/10). We fine-tuned multiple backbones (DenseNet-121, MobileNetV2, VGG16, InceptionV3, ResNet50, Xception, and a custom CNN) and evaluated the accuracy and F1-score, benchmarking against the recent literature. **Results**: On the five-class task (ALL/AML/CLL/CML/Healthy), MobileNetV2 achieved 97.9% accuracy/F1, with DenseNet-121 reaching 97.66% F1. On ALL subtypes (Benign, Early, Pre, Pro) and across tasks, DenseNet121 and MobileNetV2 were the most reliable, achieving state-of-the-art accuracy with the strongest, nucleus-centric explanations. **Conclusions**: XAI analyses (LIME, Grad-CAM) consistently localized leukemic nuclei and other cell-intrinsic morphology, aligning saliency with clinical cues and model performance. Compared with baselines, our approach matched or exceeded accuracy while providing stronger, corroborated interpretability on a substantially larger and more diverse dataset.

## 1. Introduction

**Figure 1:**
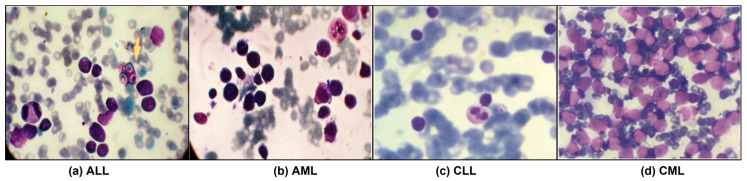
Leukemia images consisting of different sub-classes: (**a**) Acute Lymphoblastic Leukemia (ALL) [1], (**b**) Acute Myeloid Leukemia (AML) [1], (**c**) Chronic Lymphocytic Leukemia (CLL) [2], and (**d**) Chronic Myeloid Leukemia (CML) [2].

Leukemia accounts for ~8% of cancers and is classified as acute or chronic, with lymphoblastic and myeloid subtypes [1,2]. As shown in Figure 1, it is crucial to have a good understanding of the epidemiology and trends of leukemia to plan treatment accordingly [1]. Diagnosis is led by expert pathologists using peripheral blood smears and bone marrow analyses, complemented by advanced methods such as LDI-PCR, molecular cytogenetics, array-CGH, and sometimes interventional radiology [3]. These approaches are costly and time-consuming, imaging sensitivity can be influenced by genetic factors, and the disease’s complexity leaves room for diagnostic error.

Intelligent diagnostic tools are essential for precise leukemia identification. Recent advances in AI have reshaped medical imaging and hematologic analysis. Deep Learning (DL) enables rich data representation by automatically learning multi-level hierarchical features from raw medical images, starting from low-level edges and textures to high-level morphological patterns, through multiple layers of interconnected neural units that adjust their weights via backpropagation to minimize prediction error [4]. DL has enabled the automated analysis of high-dimensional medical images of a wide range of complex diseases, including cancers such as leukemia [3,5]. CNNs have proven their ability to evaluate and interpret medical images, including blood and bone marrow, facilitating accurate differentiation between various leukemia types [2,4]. XAI targets transparency by providing human-understandable rationales for model outputs and, in cancer imaging, couples modern processing and DL to both detect disease and explain decisions [6]. Because leukemia arises from acquired genetic and chromosomal abnormalities that drive malignant clonal expansion, timely and accurate diagnosis is critical for survival and appropriate therapy selection [4]. Although there are different types of leukemia classified by cell lineage and disease progression, as shown in Table 1 [1,2], prior studies vary in design and often rely on limited datasets. To address these issues, this work proposes integrating XAI with DL to improve efficiency and accuracy, distinguishing leukemic from healthy cells using extensive blood and bone-marrow image datasets. The objectives are to develop CNN-based models for AML, ALL, CML, and CLL, incorporate LIME and Grad-CAM for explainability, evaluate with Accuracy and F1-Score, and benchmark against state-of-the-art approaches [2,7,8] using transfer-learning backbones, such as DenseNet, VGG, MobileNet, Inception, ResNet, and Xception. However, these approaches are costly and time-consuming, imaging sensitivity can be influenced by genetic factors, and the disease’s complexity leaves room for diagnostic error [9].

## 2. Related Work

### 2.1. Deep Learning for Leukemia Diagnosis

#### 2.1.1. Single-Type Pipeline Classifier

**Figure 2:**
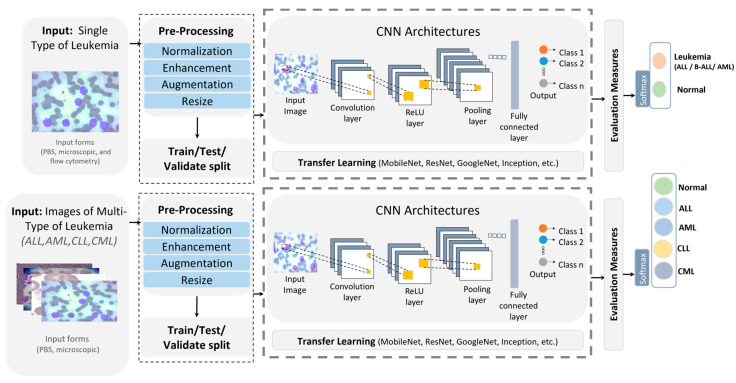
Single-type versus multi-type leukemia classification pipeline.

A growing body of work applies DL to diagnose and classify a single leukemia type, highlighting the promise of CNNs, hybrid pipelines, and ensembles in boosting accuracy [4,10,11,12]. Figure 2 illustrates a streamlined single-type versus multiple-type classifiers workflow. The Single-Type and Multiple-Type classifier pipelines are recognized and differentiated based on the number and diversity of leukemia classes targeted and the corresponding network design and labeling strategy.

- Single-Type Pipeline: Focuses on binary classification by distinguishing one leukemia type (e.g., ALL) from healthy or benign samples. It typically involves fewer image categories, simpler network heads (two output neurons), and is trained on datasets that include only one disease type.
- Multiple-Type Pipeline: Extends this to multi-class classification across several leukemia types (e.g., ALL, AML, CLL, CML, and Healthy). This pipeline uses multi-label encoding (five output neurons in our case) and is trained on a consolidated dataset where each sample is annotated according to its specific subtype.

Inputs from peripheral blood smears and microscopic images undergo normalization, enhancement, augmentation, and resizing, then the dataset is split into training and testing before a CNN performs binary classification, with standard evaluation metrics reported [4,11]. Rather than training from scratch, many studies use transfer learning, commonly MobileNet for lightweight screening and ResNet50 for feature extraction, to improve predictive performance [11,12]. Representative examples include a survey showing that CNN-based analysis of bone-marrow images is effective for ALL detection and that techniques, such as CatBoost, XGBoost, and transfer learning, further raise performance while underscoring the need for larger, more diverse datasets [4]. A B-ALL classifier built on a lightweight CNN with a tailored segmentation step reported high accuracy, with a MobileNetV2 variant performing best for early and efficient screening [11]. Another study proposed ResRandSVM, combining ResNet50 features, Random Forest feature selection, and SVM classification, achieving 90% accuracy, 90.2% precision, 95.7% recall, and a 92.9% F1-score on PBS images [12]. An ensemble that fused DenseNet-201 features with BiLSTM and GRU encoders and an MSVM head reached 96.29% accuracy, 94.58% sensitivity, 98% specificity, 96.23% F1-score, and 97.93% precision on the C-NMC 2019 dataset, demonstrating the strength of DL-sequence hybrids for ALL recognition [10].

#### 2.1.2. Multi-Type Pipeline Classifier

Recent multi-type leukemia studies report notable gains in diagnostic performance using DL methods [2,13]. Figure 2 shows a typical pipeline in which images (for ALL, AML, CLL, and CML sourced from PBS and microscopy) are normalized, enhanced, augmented, and resized, split into training and test sets, and passed to CNN-based models. Performance is then measured with standard metrics, and transfer learning is used to estimate accuracy. More blood types are investigated [2,13]. A hybrid scheme that pairs deep transfer learning feature extractors, including VGG, Xception, InceptionResV2, DenseNet, and ResNet, with ML classifiers (RF, XGBoost), was proposed for both binary and multi-class tasks on constrained datasets [2]. Using ALL-IDB for binary classification and a private set with AML, CLL, and CML for multi-class, the method reached 97.08% in multi-class classification. VGG16 and DenseNet-121 were trained to classify two leukemia types and three lymphoma subtypes, merging three public sources to cover eight cancers (including AML, ALL subtypes, CLL, FL, and CML) [13]. After resizing and augmentations, such as shear, zoom, and flips, VGG16 outperformed DenseNet-121 with 98.2% accuracy. Table 2 contrasts DL approaches targeting single versus multiple leukemia types across bone-marrow smears and PBS, spanning CNNs and hybrid designs. Pretrained backbones appearing in these works include AlexNet [4], DenseNet [2,4,10,13], MobileNet [4,11], Inception [2], Xception [2], ResNet [2,4,12] and VGG [2,13], with reported accuracies summarized therein. Despite strong results, key gaps remain: limited and noisy datasets, potential generalization issues, and opaque decision processes. XAI is therefore essential to clarify model reasoning and bolster clinical trust and reliability.

### 2.2. XAI for Leukemia Diagnosis

XAI has recently driven major gains in leukemia diagnostics by transforming deep learning models from opaque classifiers into transparent decision-support tools. Through visual explanation methods, such as Grad-CAM and LIME, researchers have verified that model attention corresponds to clinically meaningful regions, such as nuclear morphology and chromatin texture [7,8,14,15,16,17,18]. These works use backpropagation explainers, such as CAM and Grad-CAM, to make CNN decisions transparent. In [14], a pipeline combined precise WBC-nuclei segmentation (U-Net + Grad-CAM) with modified ResNet-50, delivering 0.91 segmentation accuracy and 99.9% classification across six datasets. Ref. [15] compared heatmap methods on ALL_IDB2 and found that Grad-CAM with GoogleNet best-aligned relevant pixels with key morphology: 43.61% of relevant pixels lay in diagnostic structures, with nuclei containing 73.97% of important pixels; for ALL cells, 91.90% of relevant pixels were in the nucleus versus 8.10% in cytoplasm, while healthy cells showed 54.26% in nucleus and 45.74% in cytoplasm. Ref. [16] created ALL-IDB WSI patches and proposed OrthoALLNet with Grad-CAM, achieving 96.06% accuracy. A feature-fusion ensemble of EfficientNetB7 and MobileNetV3Large with Grad-CAM reached 99.3% accuracy/F1 and 0.997 AUC across ALLIDB1, ALLIDB2, and ASH [17]. Using CAM, Grad-CAM, and Grad-CAM++ on multiple backbones, [18] reported top metrics around 94% accuracy/precision and 93% recall/F1. For multi-type detection, [7] fine-tuned VGG16 (last three conv layers) with an SVM head and Grad-CAM on a subject-independent smear dataset (expanded from 750 to 1250 images), attaining 84% accuracy. By contrast, a perturbation-based approach with LIME combined transfer-learned VGG-16 and Inception for binary ALL-IDB and a private multi-class set, yielding 83.33% (binary) and 100% (multi-class), though the latter was likely inflated by the small sample size [8]. Table 3 summarizes XAI methods (model-specific and model-agnostic) applied to leukemia, including Grad-CAM [7,14,15,16,17,18] and LIME [8], the DL backbones used, and reported performance, which ranges roughly from 68% to 99% accuracy. Despite high scores, studies often face limited or noisy data and computational overhead; XAI helps address trust and interpretability for clinical adoption.

## 3. Methodology

### 3.1. Proposed Deep Learning Models

**Figure 3:**
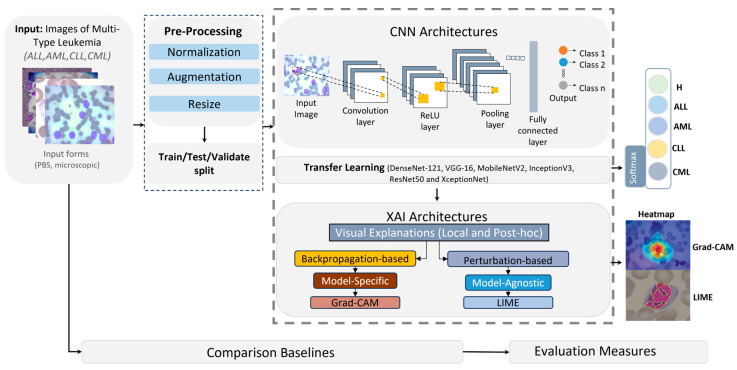
General framework of the proposed CNN and XAI model for leukemia detection.

In this section, we present an AI-driven pipeline for diagnosing leukemia from microscopic imagery spanning four disease categories: Acute Lymphoblastic Leukemia (ALL), Acute Myeloid Leukemia (AML), Chronic Lymphocytic Leukemia (CLL), and Chronic Myeloid Leukemia (CML). The approach couples convolutional neural networks (CNNs) with explainable AI (XAI) to both perform classification and expose the model’s decision rationale, thereby enhancing diagnostic accuracy and interpretability for clinical end-users. As depicted in Figure 3, the framework comprises three stages.

**Figure 4:**
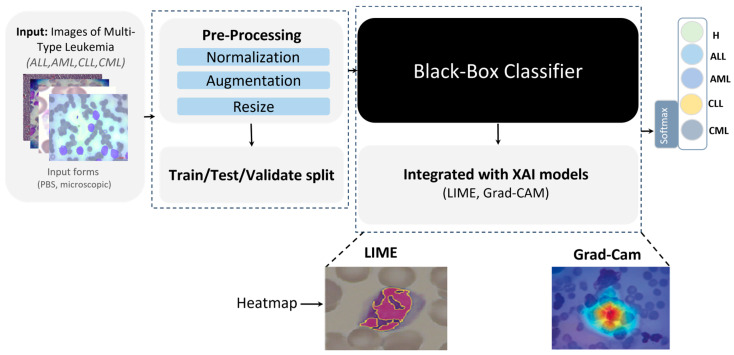
Proposed XAI approach for leukemia detection.

- Preprocessing: Input images—provided in BMP, JPG, or TIFF—were standardized via normalization, resized to 224 × 224 pixels to ensure CNN compatibility, and augmented to expand sample diversity and mitigate overfitting.
- Model training: The processed corpus was partitioned into training (80%) and testing/validation (20%) splits. We fine-tuned pretrained CNN backbones (DenseNet-121, VGG-16, ResNet50) by replacing their terminal classification layers to adapt them to leukemia subtype prediction. Deep learning was selected as the core analytical approach because microscopic leukemia diagnosis involves highly complex, high-dimensional visual patterns that cannot be effectively captured by conventional feature-engineering or shallow classifiers.
- Explainability: Post hoc XAI methods, specifically LIME and Grad-CAM, as shown in Figure 4, were applied to generate heatmaps that highlighted image regions most influential to the classifier, rendering model behavior transparent and clinically interpretable.

Our framework builds upon well-established deep learning backbones (e.g., DenseNet-121, MobileNetV2, VGG16, InceptionV3, ResNet50, Xception) and standard XAI techniques (LIME and Grad-CAM). While the proposed pipeline leverages established CNN backbones and standard XAI methods, its novelty lies in the integration and large-scale evaluation of these techniques on a unified, multi-type leukemia dataset, enabling simultaneous optimization of diagnostic performance and interpretability. Model performance is quantified using standard metrics, including accuracy and F1-score, to assess reliability across leukemia categories. Finally, we benchmark the proposed CNN + XAI pipeline against conventional deep learning baselines without XAI to demonstrate advantages in both predictive performance and interpretability.

### 3.2. Proposed XAI Techniques

Our explainability strategy combines two complementary perspectives:

- Model-agnostic (LIME)—provides local, quantitative explanations by perturbing input pixels and observing how model predictions change;
- Model-specific (Grad-CAM)—provides visual, qualitative explanations by tracing class-specific gradients back through the final convolutional layer.

#### 3.2.1. LIME

LIME provides a thorough explanation for the predictions made by any classifier or regressor by locally approximating these predictions with an interpretable model [19]. This technique is particularly useful in the field of medical image analysis, where it serves as an XAI approach designed to make the predictions of ML or DL models understandable to laypersons. LIME achieves its explanatory power by altering the feature values of a single data sample and observing the impact on the result. This process involves perturbing the input images and observing the changes in the model’s prediction, effectively pinpointing the image features that substantially impact the model’s decision [20]. An “explainer” then offers estimates for each individual data sample, detailing the contribution of each feature to a prediction for a particular sample, thereby facilitating a local level of understanding [19], which can be obtained as in [20], as follows:

(1) $$L\left(f,g,\pi_{x}\right)+\Omega \left(g\right)=\sum_{i=1}^{N}w_{i}\cdot {\left(f\left(x_{i}\right)-g\left(x_{i}^{\prime }\right)\right)}^{2}+\Omega (g)$$

where f and g represent functions with different inputs. To be specific, $f\left(x_{i}\right)$ refers to the function f applied to the original input $x_{i}$, while $g\left(x_{i}^{\prime }\right)$ refers to the function *g* applied to the perturbed input $x_{i}^{\prime }$. The input $x_{i}^{\prime }$ is a modified version of $x_{i}$ used to assess the robustness and performance of the model g. The term Ω(g) is a regularization term used to manage the complexity of g [20].

#### 3.2.2. Grad-CAM

**Figure 5:**
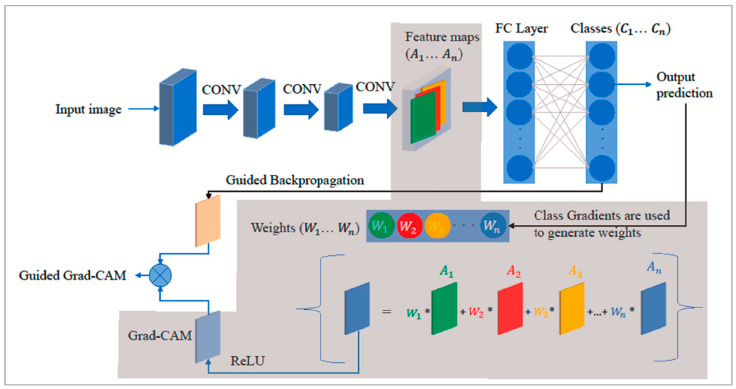
Grad-CAM (gray-shaded) architecture [21].

Grad-CAM provides visual explanations for CNN models by leveraging the high-level semantic and spatial information captured by the last CNN layer. Grad-CAM identifies the importance of each neuron for a target class using backpropagated gradients. Gradients for non-target classes are set to zero, and the non-zero signal is backpropagated to generate Rectified Convolutional Feature Maps (RCFM), which are combined to produce the Grad-CAM localization map [2]. Figure 5 shows the Grad-CAM architecture [21]. To generate this map, Grad-CAM calculated the gradient of the target class score $y^{c}$ with respect to the feature map $A^{k}$ of the convolutional layer. These gradients were aggregated across spatial dimensions using indices i and j, leading to the computation of significance weights $\alpha_{k}^{c}$ for each feature map, as follows [20]:

(2) $$\alpha_{k}^{c}=\frac{1}{Z}\sum_{i}\sum_{j}\frac{\partial y^{c}}{\partial A_{ij}^{k}}$$

where Z denotes a normalization factor that is equal to the total number of elements in the feature map. The Grad-CAM heatmap $L_{GC}^{c}$ for a specific target class c is created by combining the forward activation maps with a weighted approach, followed by the application of a ReLU function. This technique is specifically crafted to visualize only the features that have a positive impact on the class of interest, which can be obtained as in [20], as follows:

(3) $$L_{GC}^{c}=ReLU(\sum_{K}\alpha_{k}^{c}A^{k})$$

## 4. Experimental Setup

### 4.1. Datasets

**Figure 6:**
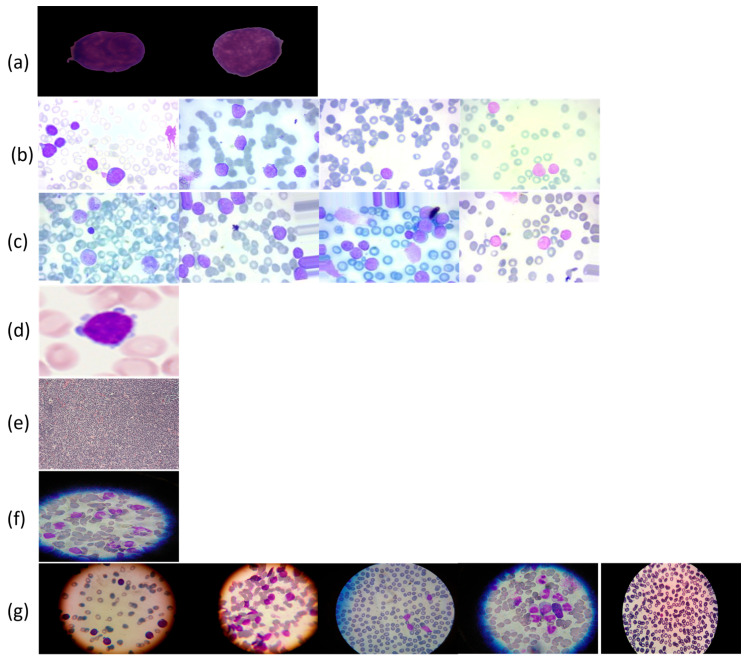
Leukemia types and subcategories: (**a**) ALL: Segmented ALL and healthy samples; (**b**) B-ALL: Benign, Early, Pre, and Pro stages; (**c**) ALL: Benign, Early, Pre, and Pro stages; (**d**) AML; (**e**) CLL; (**f**) CML; (**g**) ALL, AML, CLL, CML, and H (healthy).

To support the experimental evaluation of leukemia diagnosis, we assembled a benchmark corpus by aggregating seven publicly available datasets sourced from Kaggle and Rabinndata. The consolidated dataset integrated seven open repositories (ALL_IDB1–2, C_NMC_Leukemia, MLL, Raabin-WBC, Peripheral_Blood_Cells, BloodMNIST, and BloodCell_Images) comprising 66,550 images. All sets were curated and harmonized to yield consistent annotations that reflect the morphological variability characteristic of distinct leukemia subtypes. The consolidated benchmark encompassed Acute Lymphoblastic Leukemia (ALL), Acute Myeloid Leukemia (AML), Chronic Lymphocytic Leukemia (CLL), and Chronic Myeloid Leukemia (CML), and included healthy controls for the ALL subset, as illustrated in Figure 6. This breadth of material provided a robust foundation for deep learning-based classification and diagnostic modeling. The integrated collection comprised 15,135 microscopic images covering ALL and healthy cells, as well as 3256 peripheral blood smear (PBS) images labeled as benign or malignant. Malignant ALL cases were further stratified into Early Pre-B, Pre-B, and Pro-B to capture lymphoblast progression. An additional 20,000 ALL images—drawn from a larger archive of approximately 130,000 cancer images—were categorized into benign versus malignant. For AML, we included 10,000 PBS images capturing hallmark myeloid morphology. The CLL component consisted of two subsets totaling 113 images extracted from malignant lymphoma material. The CML portion comprised 623 microscope images acquired via a smartphone camera, providing high-detail morphology for chronic myeloid analysis. Finally, a comprehensive dataset spanning all four leukemia types plus healthy cells contributed 20,000 microscopic images covering ALL, AML, CLL, CML, and H (healthy). The ALL subset was hierarchically annotated to mirror disease trajectory: Benign denoted non-cancerous healthy cells (specific to the ALL collection), Early marked incipient leukemic presentation, Pre indicated pre-stage abnormalities suggestive of progression, and Pro represented advanced-stage leukemia cells. Across the corpus, imaging conditions varied in resolution, magnification, and clinical context. A full specification of sources, class labels, and counts is provided in Table 4.

### 4.2. Data Splitting

To prepare data for the proposed leukemia-diagnosis model, the consolidated corpus was partitioned into training, validation, and test subsets. We first applied an 80/20 split, allocating 80% for model training and the remaining 20% held out for evaluation. The held-out portion was then divided into validation and test sets, yielding 53,239 images for training, 6654 for validation, and 6657 for testing. A stratified procedure was used to preserve class proportions so that the four leukemia categories (ALL, AML, CLL, and CML) were uniformly represented across splits, ensuring reliable and consistent evaluation (Table 5).

### 4.3. Benchmark Baselines

For comparative evaluation, we adopted three baseline configurations as summarized in Table 6. The first baseline follows Ref. [2], which addresses multi-class classification across AML, CLL, and CML using transfer learning with state-of-the-art backbones (e.g., VGG, DenseNet) coupled to conventional classifiers, such as Random Forest and XGBoost. Although that work reported 100% accuracy for a binary setting, those results are excluded from our direct comparisons due to potential overfitting concerns. The second baseline is derived from Ref. [8], which proposed an ensemble of VGG16 and Inception architectures for both binary classification of ALL versus healthy and multi-class classification over AML, CLL, and CML. LIME was employed to interpret predictions and enhance explainability. While 100% accuracy was reported for both tasks, the modest size and limited diversity of the underlying dataset likely influenced these figures, underscoring the need to assess models on larger and more heterogeneous cohorts. The third baseline follows Ref. [7], which performed classification over all four leukemia types—ALL, AML, CLL, and CML—while also incorporating healthy cells for each subtype and leveraging deep transfer learning with Grad-CAM to support interpretability. This breadth renders [7] a particularly relevant benchmark. However, our dataset did not include healthy cells across all subtypes, which constrained strict one-to-one comparability with that baseline.

### 4.4. Computational Resources and Evaluation Metrics

All experiments were executed on Google Compute Engine with a single NVIDIA A100 GPU (40.0 GB VRAM), 83.5 GB system RAM, and 166.8 GB local disk. To validate the proposed leukemia-diagnosis models, we report three standard measures as in [14]. First, the training objective is categorical cross-entropy, which quantifies the divergence between predicted class probabilities and ground-truth labels. Second, accuracy measures the proportion of correctly classified instances:

(4) $$Acc=\frac{T_{P}+T_{N}}{T_{P}+T_{N}+F_{P}+F_{N}}$$

Third, the F1-score is the harmonic mean of precision and recall:

(5) $$F1=2\times \frac{Pre\times Rec}{Pre+Rec}$$

where TP, TN, FP, and FN denote true positives, true negatives, false positives, and false negatives, respectively [14].

### 4.5. Dataset Exploration

#### 4.5.1. Samples and Visualizations

**Figure 7:**
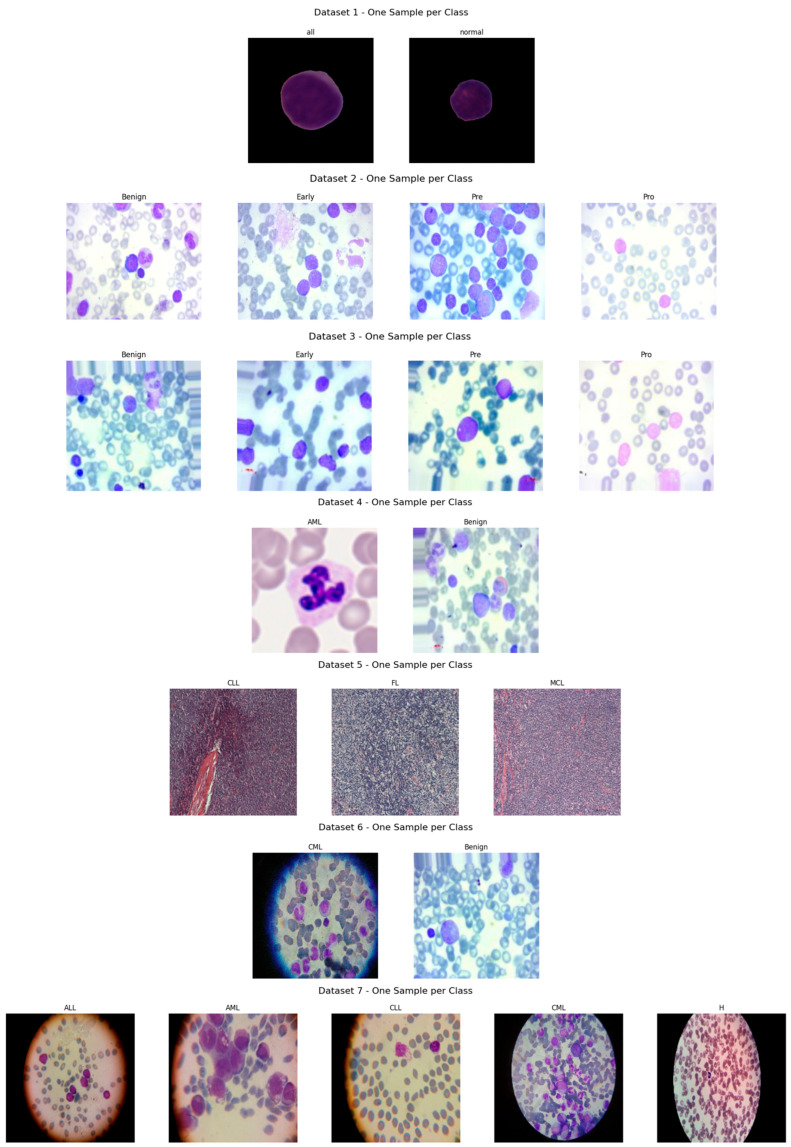
Sample microscopic images of leukemia types (ALL, AML, CLL, and CML) categorized by class and stage.

**Figure 8:**
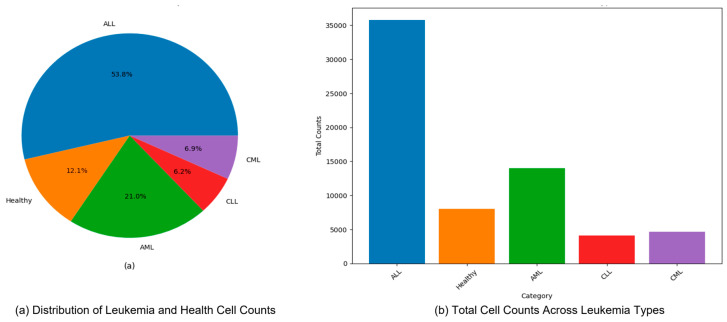
Distribution and total cell counts of leukemia types (ALL, AML, CLL, and CML) in the dataset (**a**) Distribution of Leukemia and Health Cell Counts, (**b**) Total Cell Counts Across Leukemia Types.

Figure 7 presents exemplar microscopic fields from the consolidated dataset assembled for leukemia diagnosis. The corpus covers four principal leukemia categories—ALL, AML, CLL, and CML—capturing substantial morphological variability to support fine-grained analysis and classification. We extracted representative tiles for each class, including ALL (Segmented ALL and Healthy Segmented), the B-ALL progression subclasses (Benign, Early, Pre, Pro), as well as AML, CLL, and CML. These samples illustrate intra- and inter-class heterogeneity and the presence of stage-dependent cues. Healthy controls for the ALL subset were ensured by explicitly including normal peripheral blood smear images provided within the same public ALL datasets and companion repositories, where samples are independently annotated as healthy by expert hematologists. These healthy images underwent the same preprocessing, quality control, and stratified splitting as ALL samples to ensure fair and consistent comparison. Figure 8 summarizes class representation and highlights pronounced imbalance: (a) shows proportional composition, ALL constitutes 53.8%, followed by AML at 21.0%, CML at 6.9%, CLL at 6.2%, and Healthy at 12.1%; (b) reports absolute counts, with approximately 36,000 samples for ALL, 14,000 for AML, 8000 for Healthy, 4000 for CLL, and 4500 for CML. Together, these views underscore a strong skew toward the ALL cohort and comparatively limited representation of CLL and CML.

#### 4.5.2. Training Controls to Mitigate over-/Underfitting

We control overfitting and underfitting through a combination of data, model, and training-procedure safeguards:

- Using a stratified 80/10/10 split (train/val/test), selecting all hyperparameters on the validation split only; the test split is used once, at the end, to report final metrics.
- Monitoring validation loss/F1 and stop training when validation loss does not improve for 10 epochs (patience = 10).
- Weight decay (L2 = 1 × 10^−4^) and dropout (0.3–0.5) in the classification head; label smoothing = 0.1 to reduce over-confidence.
- We evaluated four families (MixUp, AugMix, CutMix, and RandAug). MixUp (α = 0.2) and AugMix (severity = 3, width = 3) consistently minimized the train–val generalization gap and yielded the most clinically coherent explanations; results reported in the paper use those settings. (We retain RandAug/CutMix results for completeness but do not base model selection on them.)
- We tracked training vs. validation curves for loss and F1. Models are accepted only if (i) the validation F1 improves while the training F1 improves (no divergence) and (ii) the generalization gap at the chosen epoch is small (typically ≤ 2–3% for the best models). We report test metrics once for the single model checkpoint with the best validation F1.

#### 4.5.3. Preprocessing and Augmentation

**Figure 9:**
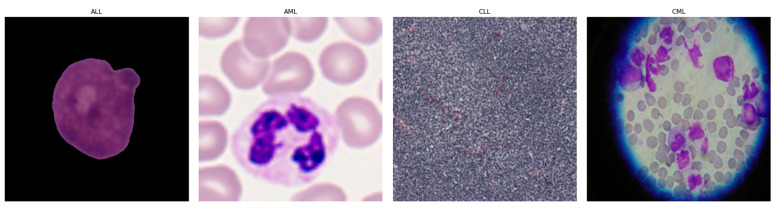
Exemplary microscopic images of ALL, AML, CLL, and CML following resizing and intensity normalization.

**Figure 10:**
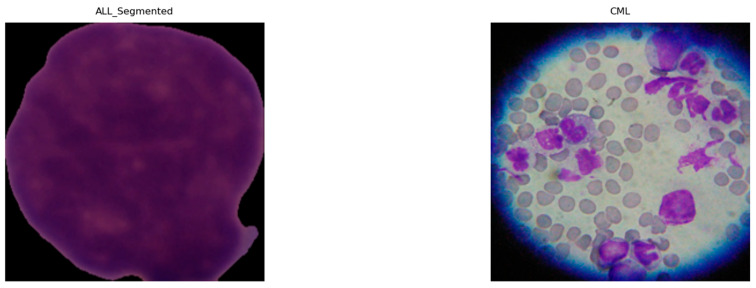
Examples of cropped ALL (segmented) and CML highlighting focused cellular regions.

Given the heterogeneous native resolutions in the source datasets (Table 5), we applied a standardized preprocessing pipeline to harmonize inputs for deep models. As shown in Figure 9, all images (ALL, AML, CLL, and CML) were resized to 224 × 224 pixels and intensity-normalized to [0, 1] to stabilize optimization and reduce variance across acquisition settings. To remove non-informative borders common in segmented or misaligned fields, we performed ROI cropping by detecting the largest contour on a binary mask and extracting its bounding box. The binary mask is used to detect the largest contour because it provides a clean, threshold representation of the cell region, enabling the precise localization of the nucleus while minimizing background interference. Figure 10 illustrates examples from ALL (Segmented) and CML, where black margins and microscope artifacts were trimmed, concentrating the field of view on diagnostically relevant cellular morphology and minimizing background noise prior to learning. Because our datasets came from multiple sources (Kaggle and Rabinndata) with varying magnifications and background noise, a standardized denoising and ROI-cropping procedure was implemented before normalization and resizing.

**Figure 11:**
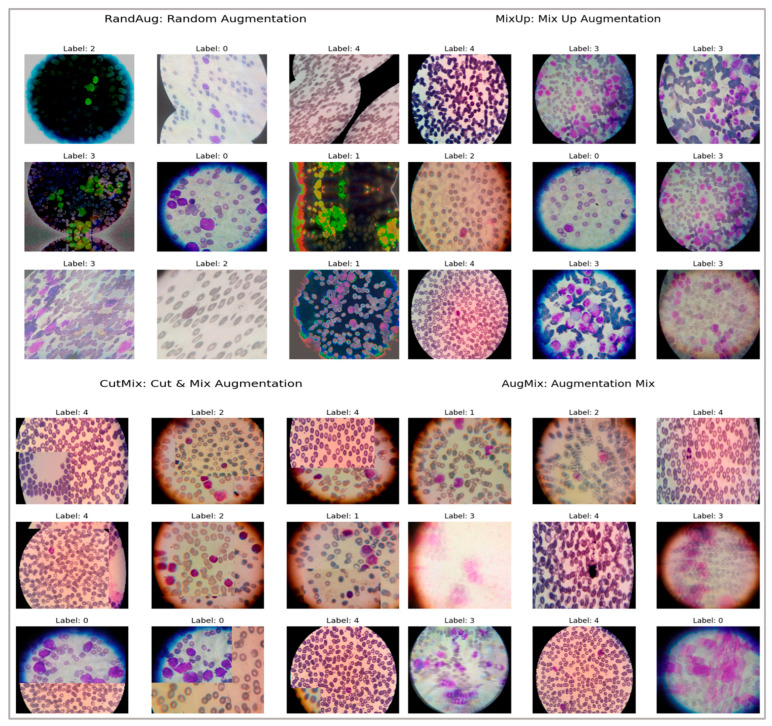
Samples of augmented ALL, AML, CML, CLL, and Healthy images to address class imbalance.

To enhance generalization and robustness, we employed a suite of augmentation strategies, exemplified in Figure 11. RandAug reduces policy search to two hyperparameters: the number and magnitude of randomly sampled transforms, which enables effective, dataset-direct augmentation with low tuning overhead [23]. MixUp forms convex combinations of images, including label pairs to encourage locally smooth decision boundaries and mitigate overfitting, albeit at the risk of producing less natural composites for fine localization tasks [24]. CutMix pastes a rectangular patch from one image into another and mixes labels proportionally to the patch area, yielding more realistic spatial structure and improved weakly supervised localization with minimal computational cost [25]. AugMix constructs short stochastic augmentation chains and enforces prediction consistency across their mixtures, substantially improving robustness under corruption and distribution shift while maintaining manageable training overhead [26].

### 4.6. Fine-Tuning Procedure

Each CNN backbone (DenseNet-121, VGG16, ResNet50, InceptionV3, Xception, and MobileNetV2) was initialized with ImageNet pretrained weights and adapted through a two-stage transfer-learning strategy. In the first stage, all convolutional layers were frozen, and only the custom classification head (Global Average Pooling → Dropout → Dense → Dropout → Softmax) was trained for 10–15 epochs using the Adam optimizer (learning rate = 1 × 10^−3^). After stabilization, the final 2–4 convolutional blocks of each backbone were unfrozen and fine-tuned end-to-end with a reduced learning rate (1 × 10^−5^) and weight decay = 1 × 10^−4^. Early stopping (patience = 10) and ReduceLROnPlateau (factor = 0.1) were applied, and MixUp/AugMix augmentations were used to enhance robustness. The best validation-F1 checkpoint was finally evaluated on the independent test set.

## 5. Experimental Results

### 5.1. Experimental Results from CNNs and Pretrained Models

We evaluated seven architectures (Xception, VGG16, ResNet50, MobileNetV2, InceptionV3, DenseNet121, and a custom CNN) under four augmentation regimes (MixUp, AugMix, CutMix, and RandAug) for binary and multiclass leukemia classification. Models were trained with a batch size of 32 for up to 100 epochs using early stopping and learning-rate scheduling. Performance is summarized in Table 6, Table 7, Table 8 and Table 9, reporting on training/validation/test splits using accuracy, loss, and F1-score. Across tasks and backbones, MixUp was the most consistently effective augmentation, typically delivering the highest F1-scores and lowest (or near-lowest) losses. AugMix was a strong runner-up, especially on MobileNetV2 and the custom CNN. CutMix often produced competitive accuracy, but frequently with higher losses, suggesting less confident predictions. RandAug was least reliable: despite occasionally yielding low losses, it often degraded the F1-score, indicating overconfident yet inaccurate predictions.

#### 5.1.1. Experiment (A)—Binary ALL vs. Healthy (Table 7)

MixUp dominated across pretrained models, e.g., Xception (Accuracy 86.10%, F1 83.32%), VGG16 (79.19%, 73.61%), ResNet50 (78.54%, 73.42%), MobileNetV2 (83.24%, 79.37%), InceptionV3 (80.80%, 77.05%), and DenseNet121 (84.94%, 81.59%). The custom CNN was an exception: CutMix yielded the best test results (Accuracy 88.95%, F1 87.75%, Loss 0.3168). AugMix was stable but generally below MixUp; RandAug underperformed (e.g., ResNet50 F1 40.41%; InceptionV3 F1 70.23%). These results can be interpreted because MixUp’s label-space smoothing reliably improved generalization, CutMix’s spatial mixing helped a shallower CNN, while RandAug’s heavy randomness likely disrupted fine cellular cues.

#### 5.1.2. Experiment (B)—Multiclass (CLL, FL, CML) (Table 7)

This lymphoma triplet was the hardest setting. Best results were achieved by DenseNet121 + MixUp (Accuracy 62.10%, F1 62.20%, Loss 0.7763). Other notable peaks include the following: MobileNetV2 + AugMix (Accuracy 59.47%, F1 58.38%, Loss 0.9422; CutMix had the lowest loss 0.9406 but lower F1 48.60%), Xception + MixUp (55.26%, 54.63%), VGG16 + CutMix (49.45%, 49.08%), and ResNet50 + MixUp (48.96%, 42.73%). The custom CNN struggled; AugMix was best for it (Accuracy 45.24%, F1 42.41%), while RandAug showed low loss but very low F1 (20.43%), indicating miscalibration. While no single augmentation dominated across all backbones, deeper networks benefited most from MixUp/CutMix.

#### 5.1.3. Experiment (C)—Multiclass CALL Subtypes (Benign, Early, Pre, Pro) Across Two Datasets (Table 8)

Performance was uniformly high, with MixUp most often on top. The subtype morphology is highly separable. Thus, MixUp generally maximizes performance, while AugMix particularly benefits MobileNetV2 and the CNN. CutMix attains strong accuracy but with a higher loss, indicating less confident predictions.

#### 5.1.4. Experiment (D)—Five-Class: (ALL, AML, CLL, CML, Healthy) (Table 10)

MixUp again led for most models: MobileNetV2 (F1 97.88%, Accuracy 97.90%, Loss 0.0775), DenseNet121 (F1 97.66%, lowest loss 0.0806), VGG16 (F1 97.29%, Loss 0.1099), and InceptionV3 (F1 95.24%). Xception peaked with AugMix (F1 96.30%, Accuracy 96.28%, Loss 0.1183), with MixUp a close second (F1 96.26%). ResNet50 lagged (MixUp F1 82.33%), and the custom CNN showed higher variance (MixUp F1 92.38%; RandAug weakest at 79.79%, Loss 0.4808). These results can be interpreted as MobileNetV2 and DenseNet121 being the most reliable across augmentations; Xception preferred AugMix in this five-class setting. RandAug frequently produced unbalanced or overconfident predictions.

**Table 10:** Performance of deep learning models on five-class leukemia classification (ALL, AML, CLL, CML, Healthy) under four augmentation strategies (AugMix, MixUp, RandAug, and CutMix); trained for up to 100 epochs with early stopping.

Model	Augmentation	Train			Val			Test		
		Acc.	Loss	F1	Acc.	Loss	F1	Acc.	Loss	F1
Xception	**AugMix**	0.9045	0.2603	0.9044	0.9637	0.1056	0.9633	**0.9628**	**0.1183**	**0.9630**
	MixUp	0.9199	0.4214	0.8737	0.9688	0.1003	0.9687	0.9622	0.1234	0.9626
	RandAug	0.8280	0.4444	0.8275	0.9304	0.1898	0.9296	0.9310	0.1929	0.9316
	CutMix	0.7079	0.8873	0.6560	0.9481	0.2301	0.9480	0.9523	0.2355	0.9525
VGG16	AugMix	0.8821	0.3157	0.8824	0.9516	0.1487	0.9510	0.9575	0.1288	0.9579
	**MixUp**	0.9168	0.4464	0.8692	0.9743	0.1065	0.9741	**0.9725**	**0.1099**	**0.9729**
	RandAug	0.8277	0.4404	0.8268	0.9269	0.1995	0.9257	0.9432	0.1892	0.9430
	CutMix	0.6885	0.9281	0.6404	0.9466	0.2502	0.9460	0.9438	0.2429	0.9440
ResNet50	AugMix	0.6714	0.8285	0.6706	0.8029	0.5207	0.8002	0.7855	0.5172	0.7848
	**MixUp**	0.7198	0.8360	0.6892	0.8271	0.4846	0.8264	**0.8215**	**0.4820**	**0.8233**
	RandAug	0.5595	1.0700	0.5579	0.7324	0.7375	0.7329	0.7105	0.7652	0.7138
	CutMix	0.5553	1.2279	0.5149	0.8054	0.6240	0.8061	0.8146	0.6363	0.8177
MobileNetV2	AugMix	0.9052	0.2588	0.9056	0.9582	0.1133	0.9576	0.9656	0.1080	0.9652
	**MixUp**	0.9228	0.4107	0.8771	0.9783	0.0823	0.9782	**0.9790**	**0.0775**	**0.9788**
	RandAug	0.8555	0.3893	0.8553	0.9355	0.1722	0.9352	0.9562	0.1459	0.9564
	CutMix	0.7187	0.8620	0.6613	0.9607	0.1843	0.9604	0.9590	0.1868	0.9595
InceptionV3	AugMix	0.8477	0.4069	0.8481	0.9340	0.1965	0.9337	0.9328	0.2166	0.9335
	**MixUp**	0.8992	0.4942	0.8531	0.9541	0.1495	0.9541	**0.9525**	**0.1668**	**0.9524**
	RandAug	0.7856	0.5631	0.7844	0.8977	0.2902	0.8976	0.8999	0.3001	0.8999
	CutMix	0.6845	0.9488	0.6349	0.9360	0.2649	0.9358	0.9302	0.2815	0.9301
DenseNet121	AugMix	0.9086	0.2477	0.9080	0.9703	0.0940	0.9702	0.9662	0.0934	0.9665
	**MixUp**	0.9221	0.4169	0.8761	0.9793	0.0743	0.9792	**0.9763**	**0.0806**	**0.9766**
	RandAug	0.8766	0.3261	0.8755	0.9597	0.1178	0.9594	0.9624	0.1183	0.9628
	CutMix	0.7027	0.8896	0.6519	0.9551	0.2096	0.9549	0.9563	0.2092	0.9565
CNN	AugMix	0.8451	0.4290	0.8431	0.8745	0.3266	0.8690	0.8761	0.3147	0.8772
	**MixUp**	0.9072	0.4809	0.8544	0.8846	0.3279	0.8833	**0.9251**	**0.2502**	**0.9238**
	RandAug	0.7689	0.6108	0.7675	0.7883	0.5145	0.7816	0.7984	0.4808	0.7979
	CutMix	0.7038	0.9079	0.6564	0.8241	0.4683	0.8215	0.8699	0.3926	0.8720

### 5.2. Comparative Analysis of Model Results for Diagnosis Problems

**Figure 12:**
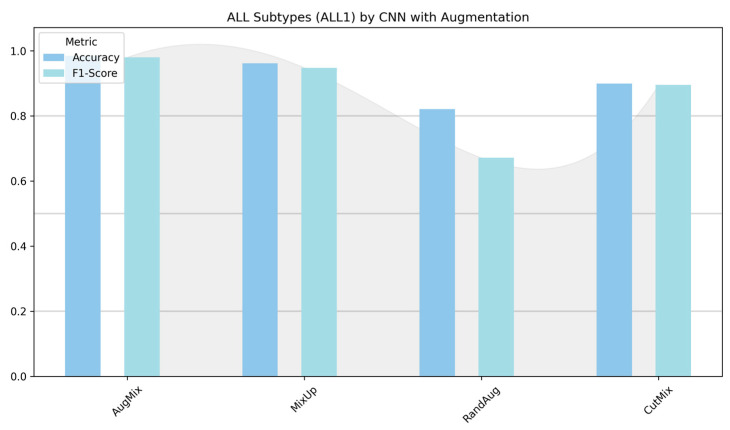

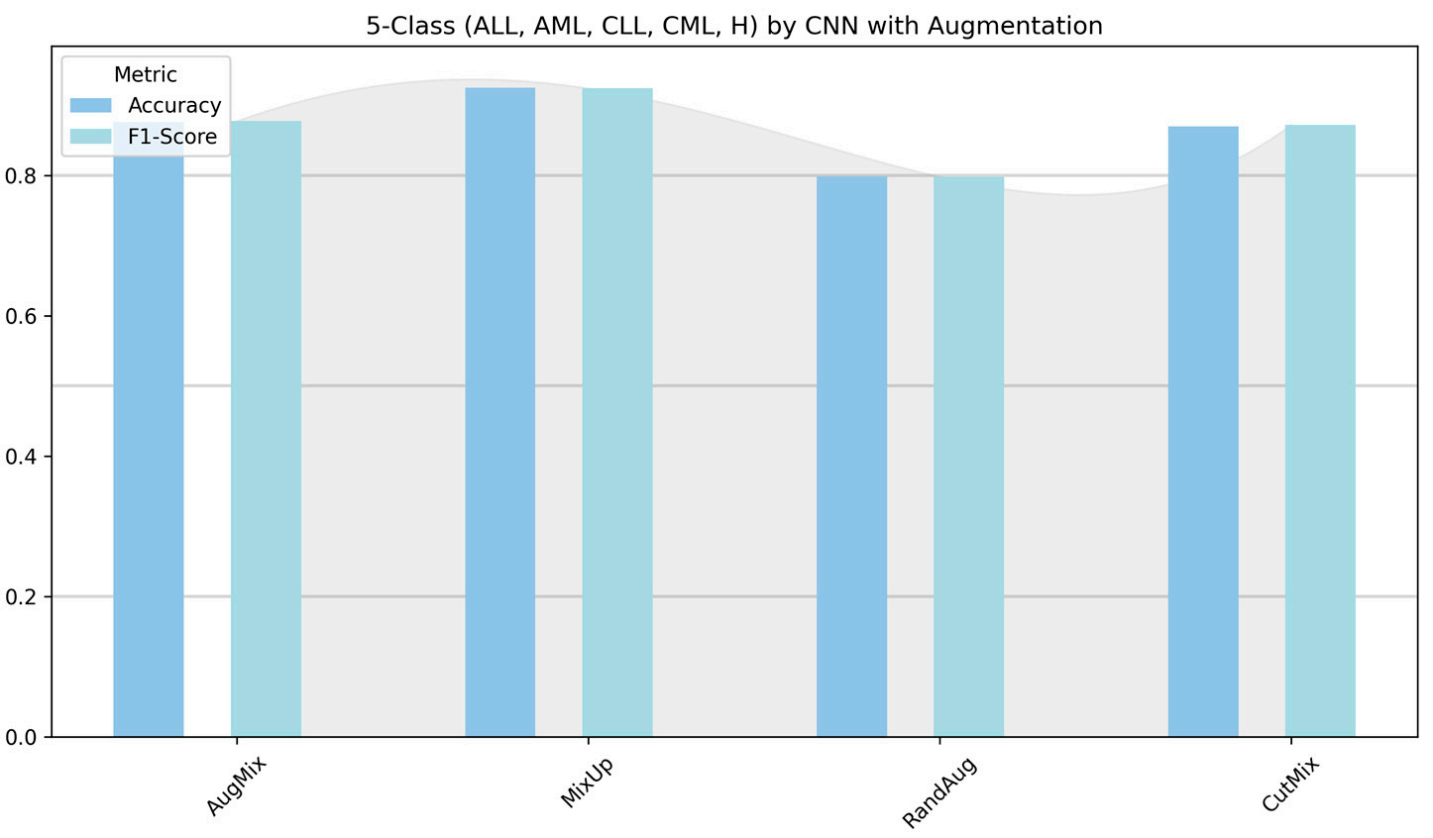
Classification performance (accuracy and F1-score) of the custom CNN for ALL subtypes (Benign, Early, Pre, and Pro) versus the five-class task (ALL, AML, CLL, CML, and Healthy).

Figure 12 contrasts the custom CNN across two settings: (left) ALL-subtype recognition (Benign, Early, Pre, Pro) and (right) the harder five-class task (ALL, AML, CLL, CML, Healthy). On the subtype dataset, the model reaches near-ceiling performance with AugMix and MixUp, while CutMix is solid but lower, and RandAug depresses F1 markedly. In the five-class experiment, absolute scores drop, reflecting greater inter-class similarity and acquisition variability; nevertheless, MixUp remains the most reliable augmentation (highest accuracy/F1), AugMix is consistently second best, CutMix is competitive but not dominant, and RandAug again lags. The CNN benefits most from augmentations that enforce smooth decision boundaries or consistency (MixUp/AugMix), whereas the heavier stochasticity of RandAug appears to disrupt fine morphological cues, which results in the gap between the easier ALL-subtype task and the more challenging five-class setting.

Augmentations such as MixUp, CutMix, and AugMix enhance model generalization by enforcing decision-boundary smoothness and prediction consistency. In these methods, linear or patch-based interpolations between images and their labels constrain the CNN to produce proportionally mixed outputs, thereby discouraging abrupt changes in class probability across the feature manifold. This mechanism aligns with the Vicinal Risk Minimization (VRM) and Manifold Mixup principles [24], which promote smoother decision surfaces and reduce overfitting. Empirically, our results (Table 6, Table 7, Table 8 and Table 9) show that such augmentations yield the highest validation F1-scores and smallest generalization gaps, confirming their effectiveness in producing robust and consistent leukemia classifiers.

**Figure 13:**
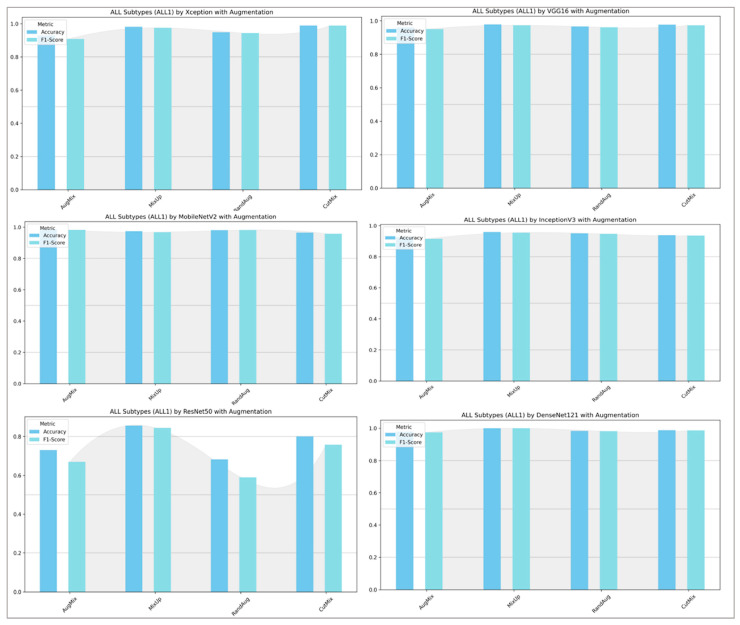
ALL-subtype classification (Benign, Early, Pre, and Pro): performance (accuracy and F1-score) of pretrained models.

**Figure 14:**
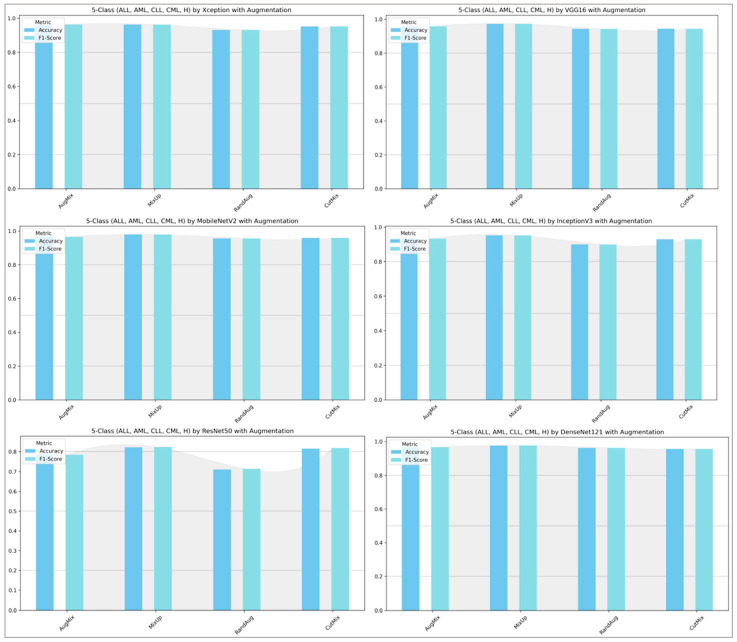
Five-class leukemia classification (ALL, AML, CLL, CML, and Healthy): performance (accuracy and F1-score) of pretrained models.

Figure 13 and Figure 14 show that augmentation choice and backbone architecture jointly govern performance. For ALL-subtype recognition (Figure 13), most pretrained models approach ceiling performance, with MixUp typically yielding the highest accuracy/F1 and AugMix close behind; RandAug is consistently weakest, and CutMix is competitive but rarely best. DenseNet121 and MobileNetV2 are the most robust, while ResNet50 exhibits the largest drop and variability. In the harder five-class setting (Figure 14), absolute scores decline slightly across models, yet the pattern persists: MixUp again dominates (especially for MobileNetV2 and DenseNet121), AugMix remains a reliable second, and RandAug trails. Notably, accuracy and F1 move in lockstep, indicating balanced precision–recall, with the biggest architecture gap seen for ResNet50 versus the stronger DenseNet121/MobileNetV2 pair.

### 5.3. Experimental Results from Explainable AI (XAI)

#### 5.3.1. Interpretability of Binary Classifiers Using LIME

**Figure 15:**
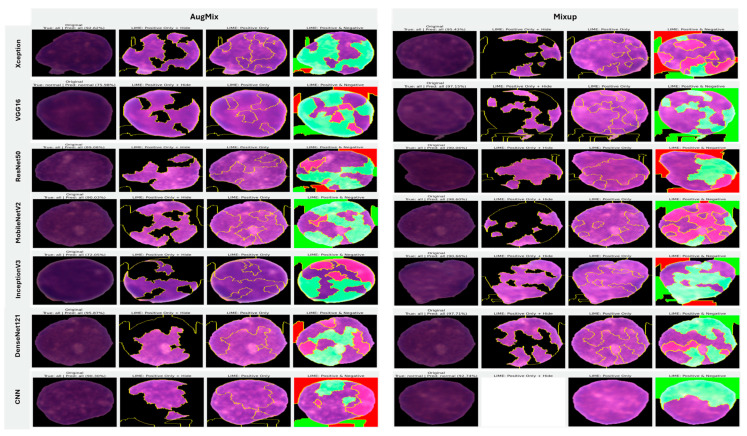

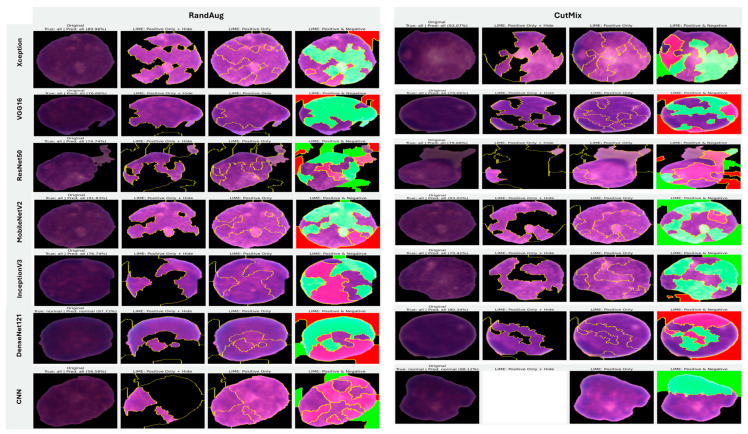
LIME-based explainability for binary ALL vs. Healthy leukemia classification, showing how the LIME explainer identifies the image regions that most strongly influence the CNN’s decision in the binary task (Acute Lymphoblastic Leukemia vs. Healthy).

In image-based tasks, LIME defines features as interpretable super-pixels obtained by segmenting the image into contiguous regions with similar color and texture, rather than individual pixels. The explainer perturbs these super-pixels by selectively masking or altering them and observes the change in model prediction to estimate each region’s local contribution to the decision. We quantified the transparency of decisions for the three binary tasks (ALL vs. Healthy, AML vs. Benign, and Benign vs. CML) by applying LIME to every trained model under four augmentation regimes (AugMix, MixUp, RandAug, and CutMix). For each model–augmentation pair, we generated three complementary explanation views—Positive Only + Hide, Positive Only, and Positive and Negative—to localize evidence supporting or opposing the predicted class. Figure 15 illustrates how the LIME explainer identifies the image regions that most strongly influence the CNN’s decision in the binary task (Acute Lymphoblastic Leukemia vs. Healthy).

- Green-highlighted super-pixels represent areas that positively contribute to the predicted class (supporting evidence).
- Red-highlighted regions denote areas that contradict the prediction or carry lower diagnostic relevance.

In the ALL samples, LIME consistently highlights the nuclear region, chromatin density, and irregular cytoplasmic boundaries—key morphological cues of leukemic cells—while in healthy cells, attention focuses on uniform nuclei with smooth borders. Across tasks, MixUp produced the most clinically coherent attributions and the strongest alignment between predictive performance and explanation quality. In the representative ALL vs. Healthy setting, Xception (F1 = 83.32%) yielded green saliency tightly concentrated on leukemic nuclei with non-relevant background suppressed (red), indicating confident, well-localized reasoning; DenseNet121 (F1 = 81.59%) and InceptionV3 (F1 = 77.05%) showed similar nucleus-centric maps. MobileNetV2 also produced focused explanations under MixUp (F1 = 79.37%) and remained competitive with CutMix (F1 = 76.93%). By contrast, RandAug routinely degraded both accuracy and interpretability. ResNet50 under RandAug (F1 = 40.41%) exhibited diffuse, noisy saliency with poor correspondence to cellular structures; switching to CutMix improved F1 to 72.30% and modestly tightened attributions, though heatmaps remained scattered. VGG16 showed comparatively better localization under RandAug (F1 = 73.95%), but its explanatory fidelity varied more across augmentations than MixUp-driven models. The custom CNN illustrates a performance–explanation tension: while CutMix (F1 = 87.75%) and MixUp (F1 = 80.64%) delivered strong scores, Positive Only + Hide maps were occasionally unreliable (partially or fully blank), suggesting weaker feature localization despite favorable metrics. A similar, albeit milder, pattern appeared with InceptionV3, which achieved solid F1 with MixUp but sometimes produced less sharply focused heatmaps.

Taken together, these results show a clear contrast between augmentation strategies: MixUp (and, secondarily, CutMix) tends to yield anatomically plausible, nucleus-focused attributions that track improvements in F1, whereas RandAug often produces overconfident yet poorly localized explanations. Notably, high accuracy does not invariably guarantee faithful localization (custom CNN, InceptionV3), underscoring the necessity of multi-view LIME diagnostics to validate decision reliability beyond aggregate performance metrics (Figure 15).

#### 5.3.2. Interpretability of Multi-Class Classifiers Using LIME

**Figure 16:**
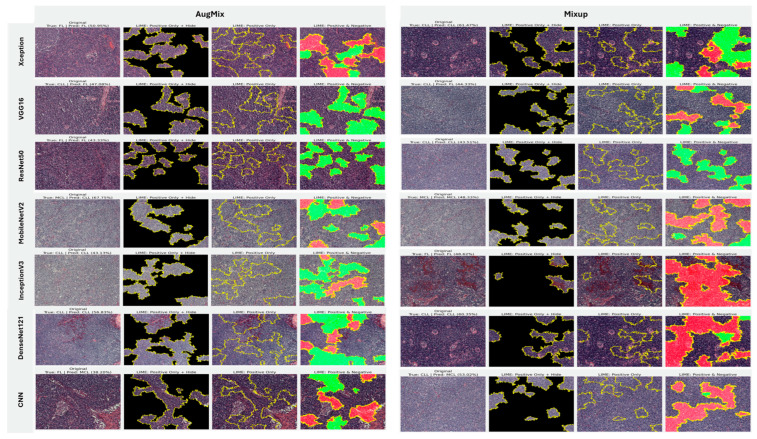

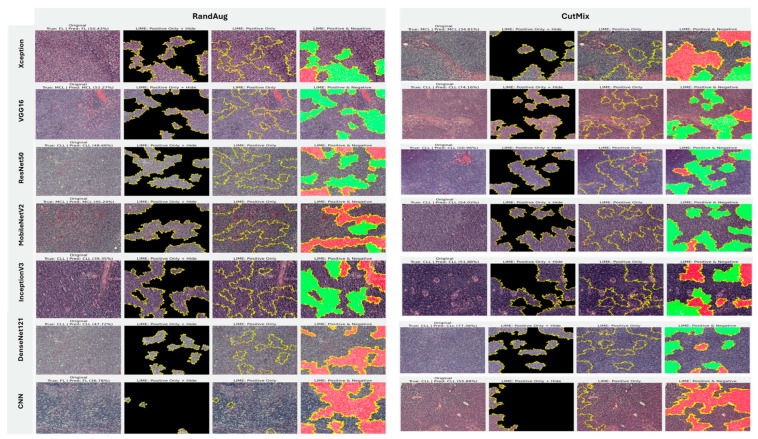
LIME visualizations for the three-class CLL–FL–CML task within our leukemia classification pipeline.

**Figure 17:**
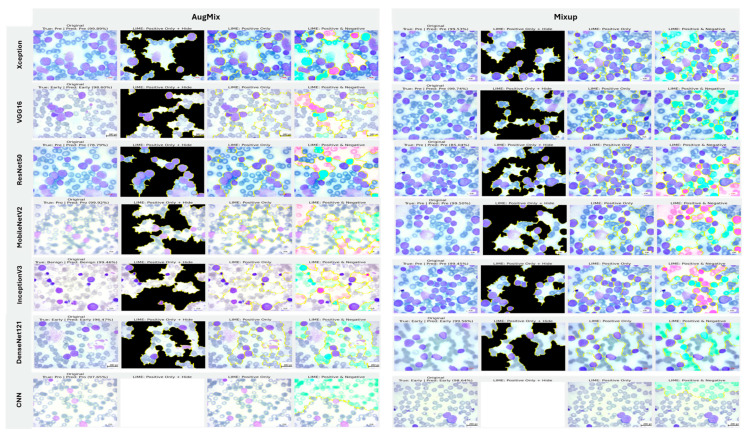

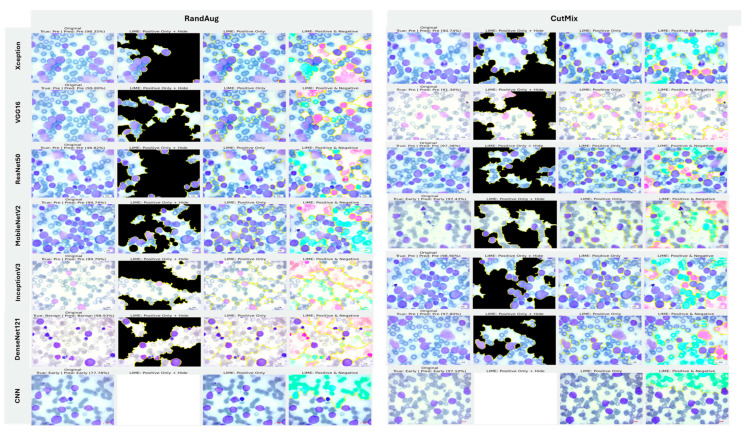
LIME visualizations for ALL-subtype leukemia classification (Benign, Early, Pre, and Pro).

**Figure 18:**
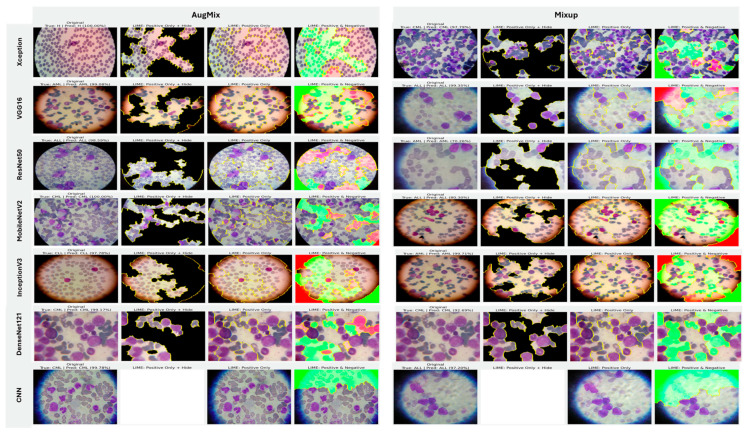

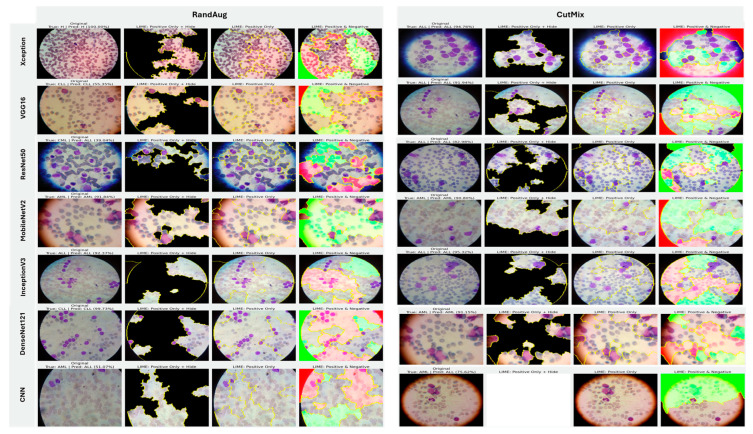
LIME visualizations for five-class leukemia classification (ALL, AML, CLL, CML, and Healthy).

Across the three multi-class settings, LIME visualizations (Figure 16, Figure 17 and Figure 18) broadly track model accuracy: clearer, nucleus-centric attributions accompany higher F1, whereas diffuse or unfocused maps coincide with weaker performance. The lymphoma triplet (CLL/FL/CML) is the most challenging—most backbones show low F1 with scattered saliency; the custom CNN is especially vague, while Xception improves under MixUp with class-relevant highlights. By contrast, ALL-subtype recognition yields strong and well-localized explanations for Xception, VGG16, MobileNetV2, DenseNet121, and InceptionV3, with DenseNet121 achieving the most consistent, sharply focused maps; the CNN, despite high scores under AugMix/MixUp, sometimes presents nearly blank “Positive Only + Hide” views, revealing an accuracy–interpretability gap. In the five-class task, DenseNet121 again leads with tight, diagnostically coherent heatmaps, closely followed by MobileNetV2; Xception and VGG16 remain competitive with spatially meaningful attributions, whereas InceptionV3’s maps are reliable under MixUp but less precise under RandAug/AugMix. ResNet50 and the CNN lag, often producing scattered or coarse saliency. The experimental results reveal that MixUp delivers the most faithful and stable explanations across models, AugMix is a solid second, while CutMix and RandAug most often degrade the interpretability.

#### 5.3.3. Interpretability of Binary Classifiers Using Grad-CAM

**Figure 19:**
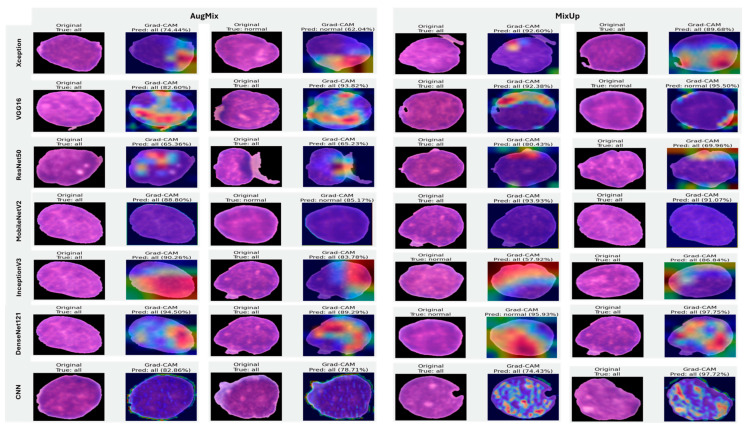
Grad-CAM visualizations for binary ALL vs. Healthy leukemia classification. Heatmaps highlight the discriminative regions driving each prediction, enabling verification that models attend to leukemic nuclei and other cell-intrinsic morphology rather than background artifacts.

Guided by the preceding experiments, we report Grad-CAM results exclusively for MixUp and AugMix, as these augmentations consistently produced the most faithful and stable explanations across architectures, whereas CutMix and RandAug generally degraded interpretability. Figure 19 presents Grad-CAM visualizations for the ALL vs. Healthy classification, providing further insights into each model’s ability to localize class-relevant regions. With regard to Grad-CAM (ALL vs. Healthy), across backbones, MixUp (right panels) produces cleaner, nucleus-centric saliency than AugMix (left), which is often broader and more diffuse. DenseNet121 and MobileNetV2 show the most clinically plausible maps; the heat strongly concentrates on leukemic nuclei with background largely suppressed, followed by Xception and VGG16, whose MixUp explanations are also well localized. InceptionV3 improves under MixUp but still exhibits occasional spillover into cytoplasm. ResNet50 remains inconsistent, with edge- or background-biased activations, especially under AugMix. The custom CNN yields the least stable attributions (noisy/perimeter-focused) under both regimes, though MixUp narrows attention somewhat. MixUp enhances both confidence and interpretability, while AugMix more commonly dilutes focus on the diagnostically relevant regions.

#### 5.3.4. Interpretability of Multi-Class Classifiers Using Grad-CAM

**Figure 20:**
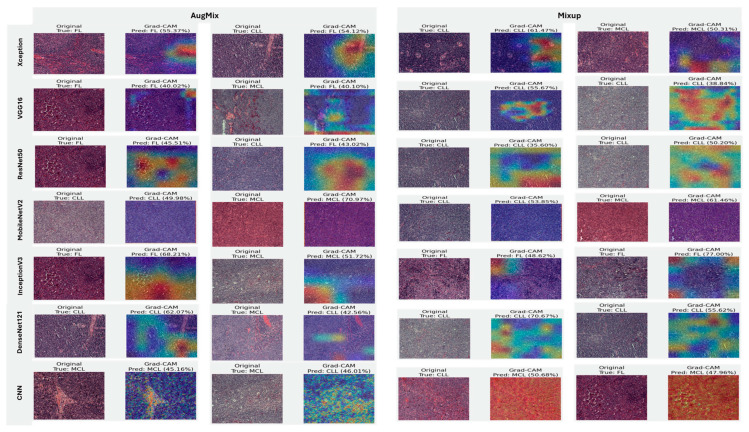
Grad-CAM visualizations for three-class leukemia classification (CLL vs. FL vs. CML).

**Figure 21:**
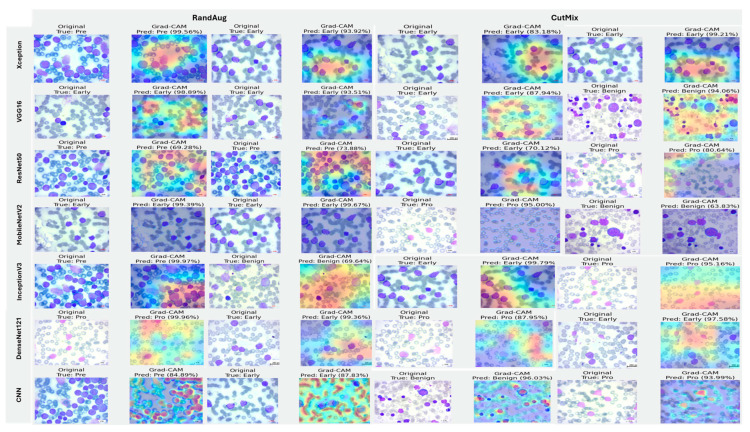
Grad-CAM visualizations for ALL-subtype leukemia classification (Benign, Early, Pre, and Pro).

**Figure 22:**
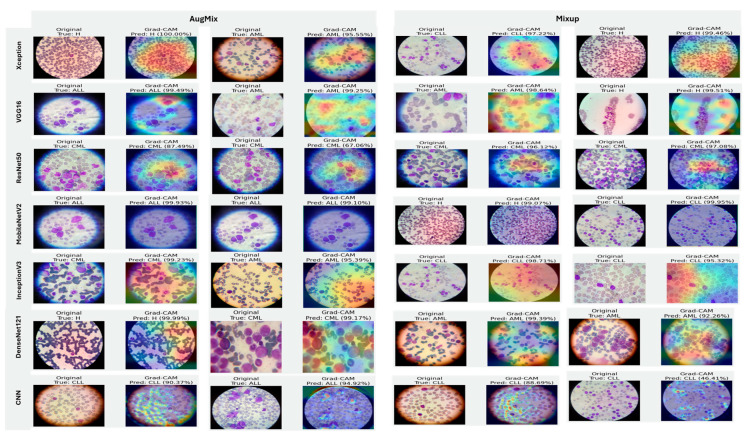
Grad-CAM visualizations for five-class leukemia classification (ALL, AML, CLL, CML, and Healthy). Saliency overlays identify class-specific cellular structures across heterogeneous samples.

Figure 20, Figure 21 and Figure 22 summarize Grad-CAM evidence across the three multi-class settings and reveal a consistent link between performance and interpretability. In the lymphoma triplet (CLL–FL–CML, shown in Figure 20), saliency is comparatively diffuse and sometimes edge-biased, especially for ResNet50 and the custom CNN—reflecting the task’s higher class similarity; MixUp generally sharpens attention over subtype-relevant tissue regions, with DenseNet121 and InceptionV3 producing the most coherent maps. In contrast, ALL-subtype recognition (Benign, Early, Pre, and Pro; Figure 21) yields compact, nucleus-centric activations across backbones, with MixUp (and closely, AugMix) focusing on diagnostically meaningful structures; DenseNet121 and MobileNetV2 are particularly well localized, whereas ResNet50 and the CNN remain less stable. The five-class setting (ALL, AML, CLL, CML, and Healthy; Figure 22) falls between these extremes: MixUp again delivers the cleanest, class-specific attributions for DenseNet121, MobileNetV2, Xception, and VGG16, while weaker models exhibit broader, less discriminative heatmaps.

Across tasks, DenseNet121 and MobileNetV2 are the most reliable, achieving state-of-the-art accuracy with the strongest, nucleus-centric explanations. DenseNet121 attains perfect F1 on the ALL-subtype dataset and ~97.7% F1 on the five-class problem, while MobileNetV2 reaches ~97.9% accuracy/F1 in the same five-class setting; in both cases, LIME and Grad-CAM concentrate on leukemic nuclei and other cell-intrinsic structures, indicating faithful attribution.

### 5.4. Comparison of Baseline Methods and the Proposed Model

Compared with prior XAI baselines, our system attains high accuracy on a larger and harder benchmark while providing richer, corroborated interpretability. Using 20,000 images spanning five classes (ALL, AML, CLL, CML, Healthy), our MobileNetV2-based model with LIME and Grad-CAM achieves 97.9% accuracy, as can be seen in Table 11. In contrast, [2] reported 100% on ALL and 97.08% on (AML, CLL, CML) using 889 ALL-IDB images plus a small private set and no XAI; results that are impressive but plausibly inflated by limited data and the absence of a Healthy class.

Baselines with XAI on smaller corpora underperform: 84% with VGG16 + SVM and Grad-CAM on 1250 AIIMS Patna images in [7], and 83.33% on ALL (ALL-IDB) using LIME in [8], with a reported 100% on a private multi-class set that again likely reflects dataset constraints. Our model matches or exceeds the multi-class performance from [2] while substantially outperforming [7] and [8] on public data, and crucially delivers complementary, cell-centric explanations (LIME + Grad-CAM) that validate attention to leukemic nuclei and support clinical trust across a far more diverse dataset.

## 6. Conclusions and Future Work

This study demonstrates the transformative potential of deep learning for leukemia diagnostics by enabling early, accurate, and automated classification of hematologic subtypes from microscopy. We engineered a CNN and XAI framework that adapts powerful pretrained backbones, namely DenseNet-121, MobileNetV2, VGG16, InceptionV3, ResNet50, and Xception, to classify multiple leukemia types (ALL, AML, CLL, CML) and healthy cells using standardized, ROI-focused images. Comprehensive evaluations across binary and multiclass settings show state-of-the-art performance: on the five-class task, MobileNetV2 reached 97.9% F1, while DenseNet-121 achieved ~97.66% F1. The lymphoma triplet (CLL vs. FL vs. CML) remained the most challenging, where DenseNet-121 attained the best scores (62.1% accuracy/62.2% F1). In binary settings, outcomes depended on the model and setup: the custom CNN achieved 88.95% accuracy on ALL vs. Healthy. Crucially, integrating XAI mitigated model opacity. LIME and Grad-CAM supplied complementary evidence maps that aligned predictions with cell-intrinsic morphology. DenseNet-121, InceptionV3, and Xception produced the most consistently nucleus-centric Grad-CAM saliency, whereas MobileNetV2 showed mixed Grad-CAM heatmaps, underscoring that high accuracy does not always guarantee faithful localization. Our approach matched or exceeded prior non-XAI baselines and substantially outperformed XAI baselines trained on smaller corpora, while offering stronger, corroborated interpretability.

The work has some limitations. The corpus is class-imbalanced (over-representation of ALL and limited CLL/CML), heterogeneous in acquisition (stains, magnification, devices), and lacks healthy controls for all subtypes. Explanations were assessed with post hoc methods that provide no formal guarantees of faithfulness. Prospective clinical validation and workflow integration were beyond scope.

Future work will (i) expand to multi-center, class-balanced cohorts with standardized staining and metadata to reduce domain shift; (ii) explore self-supervised and foundation-model pretraining, stain/style normalization, domain generalization, and calibration/uncertainty quantification (e.g., conformal risk control) to improve robustness; (iii) adopt hierarchical and multi-instance learning to model patient-level decisions from fields of view and whole-slide images; (iv) enrich explainability with complementary techniques (Integrated Gradients, SHAP, counterfactuals) and quantitative faithfulness tests, coupled with user studies of pathologist trust and decision impact; (v) implement source-disjoint and patient-level splits to eliminate potential data leakage; (vi) conduct statistical significance testing for comparative results across architectures and augmentations and evaluate deployment aspects, such as runtime constraints, on-device inference, and continuous learning/active labeling. Collectively, these directions aim to translate accurate, interpretable leukemia AI from retrospective benchmarks to prospective, real-world clinical settings.

## Figures and Tables

**Table 1:** Major types of leukemia classified by cell lineage and disease progression, showing the affected cell type, rate of development, predominant age group, and key distinguishing features for each form.

Category	Cell Type Affected	Disease Speed	Typical Age Group	Key Features
ALL	Lymphoid (immature B/T cells)	Acute	Children	Rapid onset; immature lymphoblasts
AML	Myeloid (precursor cells)	Acute	Adults	Myeloblast accumulation; cytogenetic variants
CLL	Lymphoid (mature B cells)	Chronic	Older adults	Slow progression; high lymphocyte count
CML	Myeloid (mature myeloid cells)	Chronic	Adults (50+)	Philadelphia chromosome; triphasic course

**Table 2:** Comparison of single-type vs. multi-type classifiers in the literature.

Type	Images	Type of Data	Method	Pre-Trained Models	Acc.	Limitations	Ref.
Single-Type Classifiers	-	bone marrow smear	CNN	AlexNet, DenseNet121, MobileViTv2, ResNet-50, ResNext101_32x8d, ResNext50_32x4d	88.5%	Limited datasets. Lack of external validation. Computational complexity.	[4]
	3242	PBS	CNNs	MobileNetV2	100%	Limited datasets.	[11]
	10,661	PBS	ResRandSVM	ResNet50	90%	Complex model architecture complicates interpretation.	[12]
	12,528	Microscopic	CNN-GRU-BiLSTM-MSVM	DenseNet-201	96.29%	-	[10]
Multi-Type Classifiers	889	Microscopic	Hybrid DL-ML	VGG, Xception, InceptionResNet, DenseNet, ResNet	100% for (ALL)	Limited datasets.	[2]
					97.08% for AML, CLL, CML		
					92.2% AML vs. ALL cases		
	3679	Microscopic	CNNs	VGG16	98.2%	Limited datasets. Difficulty in interpreting and explaining DL predictions.	[13]
				DenseNet-121	98.1%		

**Table 3:** Summary of visual explainable AI (XAI) techniques applied to leukemia classification, showing the model types, pre-trained models, XAI methods, performance, and limitations.

XAI Type	Model Type				Images	Model	Pre-Trained Models	XAI Model	Acc.	Limitation	Ref.
	Local/Global	Intrinsic/Post Hoc	Specific/Agnostic								
Visual: Backpropagation-based	Local	Post hoc	Model-Specific	ALL	2823	CNN	ResNet-50	Grad-Cam	99.9%	Time-consuming. Limited datasets.	[14]
	Local	Post hoc	Model-Specific	ALL	260	CNN	VGG-19, ResNet18, ResNet50, GoogleNet	Grad-Cam	-	Limited datasets.	[15]
	Local	Post hoc	Model-Specific	ALL	108	OrthoALLNet	ResNet18, ResNet34	Grad-Cam	95.91% to 96.06%	Limited datasets.	[16]
	Local	Post hoc	Model-Specific	ALL, AML	498	CNN	EfficientNetB7, MobileNetV3	Grad-Cam	99.3%	Limited datasets.	[17]
	Local	Post hoc	Model-Specific	ALL	15,114	CNN	VGG, ResNet, EfficientNet, DenseNet121, ViT, InceptionV3	CAM, Grad-CAM & GradCAM++	68% to 94%	-	[18]
	Local	Post hoc	Model-Specific	ALL, AML, CLL, CML	1250	CNN	VGG16	Grad-Cam	84%	Limited datasets.	[7]
Visual: Perturbation-based	Local	Post hoc	Model-Agnostic	ALL, AML, CLL, CML	889	CNN	VGG-16 and InceptionV3	LIME	83.33% (ALL)	-	[8]
									100% (AML, CLL, CML)	Limited datasets cause 100%	

**Table 4:** Summary of data sources of types of leukemia utilized in this work.

No.	Dataset	Type	Images	Description	Magnification	Source
1.	ALL	Microscopic	15,135	Cells segmented from 15,135 microscopic images divided into healthy and ALL.	N/A	Kaggle
2.	ALL	PBS	3256	Cells segmented from 3256 PBS images divided into two classes: benign and malignant. The ALL group with three subtypes of malignant lymphoblasts: Early Pre-B, Pre-B, and Pro-B ALL.	100×	Kaggle
3.	ALL	N/A	20,000	Contains 130,000 images of 8 types of cancer, and 20,000 images for ALL, divided into two classes: benign and malignant. The ALL group with three subtypes of malignant lymphoblasts: Early_ ALL, Pre_ ALL, and Pro_ALL.	512 × 512 pixels	Kaggle
4.	AML	PBS	10,000	Cells segmented from 10,000 PBS images from patients diagnosed with AML.	64 × 64 pixels	Kaggle
5.	CLL	N/A	113	Contains 5400 images of 3 types of malignant lymphomas, and 113 images for CLL.	N/A	Kaggle
6.	CML	Microscope	623	Contains 623 microscopic images of CML, and the images taken by smartphone camera.	N/A	Raabindata
7.	ALL, AML, CLL, CML, H	Microscope	20,000	Contains 20,000 microscopic images of ALL, AML, CLL, CML, and H (healthy).	N/A	[22]

**Table 5:** Distribution of data across training and testing datasets used in this study.

Leukemia Type	Images	Training (80%)	Validation (10%)	Testing (10%)	Image Size	Image Format
ALL	35,814	28,651	3581	3582	(450, 450), (600, 600), (224, 224), (512, 512), (640, 640)	BMP, JPEG
AML	10,000	8000	1000	1000	(64, 64)	TIFF
CLL	113	90	11	12	(1388, 1040), (512, 512)	TIFF, JPEG
CML	623	498	62	63	(4160, 3120)	JPEG
ALL, AML, CLL, CML, H	20,000	16,000	2000	2000	(1024, 768)	JPEG
Total:	66,550	53,239	6654	6657		

**Table 6:** Comparison of the measures of the proposed model with the related studies.

Ref.	Images	Dataset/Repository	Type of Leukemia	Method	XAI	Accuracy
**[2]**	889	ALL-IDB	ALL, AML, CLL, CML	VGG, Xception, InceptionResV2, DenseNet, ResNet with RF and XGBoost	-	100% for ALL
		Private dataset (AML, CLL, CML)				97.08% for (AML, CLL, CML)
**[7]**	1250	AIIMS Patna	ALL, AML, CLL, CML	VGG16 with SVM	Grad-CAM	84%
**[8]**	889	ALL-IDB	ALL, AML, CLL, CML	VGG-16 and InceptionV3	LIME	83.33% (ALL)
		Private dataset (AML, CLL, CML)				100% (AML, CLL, CML)

**Table 7:** Performance of deep learning models on binary ALL vs. Healthy leukemia classification under four augmentation strategies (AugMix, MixUp, RandAug, and CutMix); trained for up to 100 epochs with early stopping.

Model	Augmentation	Train			Validation			Test		
		Acc.	Loss	F1	Acc.	Loss	F1	Acc.	Loss	F1
Xception	AugMix	0.7372	0.5607	0.6667	0.8085	0.4230	0.7591	0.8134	0.4275	0.7698
	**MixUp**	0.8194	0.4244	0.7648	0.8574	0.3367	0.8268	**0.8610**	**0.3613**	**0.8332**
	RandAug	0.7079	0.5842	0.6272	0.7965	0.4595	0.7568	0.7706	0.4616	0.7290
	CutMix	0.7191	0.5779	0.6305	0.8301	0.4060	0.7808	0.8185	0.4112	0.7596
VGG16	AugMix	0.7242	0.5872	0.6532	0.7853	0.4834	0.7012	0.7789	0.4773	0.6958
	**MixUp**	0.7511	0.5600	0.6800	0.8045	0.4514	0.7548	**0.7919**	**0.4577**	**0.7361**
	RandAug	0.7237	0.5692	0.6353	0.7869	0.4875	0.7395	0.7711	0.5162	0.7204
	CutMix	0.7146	0.5904	0.6104	0.7965	0.4671	0.7433	0.7881	0.4755	0.7296
ResNet50	AugMix	0.7134	0.5978	0.6375	0.7732	0.5041	0.7059	0.7648	0.4989	0.6896
	**MixUp**	0.7393	0.5647	0.6677	0.7837	0.4792	0.7230	**0.7854**	**0.4805**	**0.7342**
	RandAug	0.6757	0.6309	0.5189	0.6755	0.5990	0.4055	0.6728	0.5969	0.4041
	CutMix	0.6943	0.6220	0.5978	0.7804	0.4910	0.7210	0.7812	0.4982	0.7230
MobileNetV2	AugMix	0.7386	0.5485	0.6742	0.7997	0.4480	0.7405	0.8109	0.4353	0.7518
	**MixUp**	0.8038	0.4635	0.7421	0.8165	0.4086	0.7740	**0.8324**	**0.3965**	**0.7937**
	RandAug	0.7080	0.5946	0.6358	0.7788	0.4785	0.7058	0.7837	0.4703	0.7105
	CutMix	0.7096	0.5929	0.6121	0.7989	0.4534	0.7422	0.8162	0.4414	0.7693
InceptionV3	AugMix	0.7388	0.5447	0.6708	0.7917	0.4658	0.7377	0.7874	0.4744	0.7371
	**MixUp**	0.7876	0.4839	0.7354	0.8213	0.4148	0.7857	**0.8080**	**0.4256**	**0.7705**
	RandAug	0.7164	0.5732	0.6314	0.7612	0.4938	0.7072	0.7556	0.5236	0.7023
	CutMix	0.6987	0.6060	0.6043	0.8061	0.4448	0.7478	0.8006	0.4548	0.7397
DenseNet121	AugMix	0.7597	0.5124	0.7031	0.8237	0.4050	0.7827	0.8303	0.4066	0.7882
	**MixUp**	0.8245	0.4343	0.7702	0.8518	0.3435	0.8222	**0.8494**	**0.3437**	**0.8159**
	RandAug	0.7546	0.5230	0.6898	0.8221	0.4041	0.7808	0.7903	0.4670	0.7460
	CutMix	0.7415	0.5527	0.6576	0.8462	0.3748	0.8063	0.8420	0.3870	0.8017
CNN	AugMix	0.7763	0.4865	0.7155	0.7652	0.4873	0.7521	0.7910	0.4775	0.7718
	MixUp	0.8530	0.3904	0.7997	0.8293	0.3722	0.8179	0.8178	0.3858	0.8064
	RandAug	0.7685	0.5081	0.6852	0.7139	0.5307	0.7078	0.7825	0.4952	0.7643
	**CutMix**	0.7984	0.4842	0.7259	0.8838	0.3104	0.8698	**0.8895**	**0.3168**	**0.8775**

**Table 8:** Performance of deep learning models on three-class lymphoma classification (CLL vs. FL vs. CML) under four augmentation strategies (AugMix, MixUp, RandAug, and CutMix); trained for up to 100 epochs with early stopping.

Model	Augmentation	Train			Validation			Test		
		Acc.	Loss	F1	Acc.	Loss	F1	Acc.	Loss	F1
Xception	AugMix	0.3798	1.2851	0.3778	0.4375	1.2137	0.2897	0.3998	1.1012	0.1939
	**MixUp**	0.7115	0.7518	0.6867	0.5625	1.1341	0.5369	**0.5526**	1.0876	**0.5463**
	RandAug	0.3796	1.3432	0.3756	0.3438	1.2161	0.1746	0.4420	**1.0829**	0.2776
	CutMix	0.4618	1.2466	0.4459	0.5312	1.0242	0.5106	0.4107	1.1147	0.4070
VGG16	AugMix	0.4637	1.2660	0.4558	0.4062	1.3110	0.1926	0.4261	1.0649	0.2479
	MixUp	0.4735	1.1503	0.4562	0.5000	1.0240	0.4362	0.4633	1.0887	0.4439
	RandAug	0.3570	1.4211	0.3555	0.2812	1.2176	0.2046	0.3368	1.1460	0.1916
	**CutMix**	0.4771	1.2294	0.4618	0.6250	0.9506	0.5842	**0.4945**	**0.9722**	**0.4908**
ResNet50	AugMix	0.4849	1.1454	0.4789	0.5000	1.0868	0.3810	0.3318	1.1590	0.1657
	**MixUp**	0.4794	1.2549	0.4717	0.4688	1.0313	0.3810	**0.4896**	1.0116	**0.4273**
	RandAug	0.3991	1.2831	0.3961	0.4375	1.0661	0.3206	0.3001	1.1364	0.1538
	CutMix	0.3689	1.5108	0.3515	0.4062	1.1547	0.3632	0.4633	**0.9963**	0.4008
MobileNetV2	**AugMix**	0.4538	1.1880	0.4525	0.5000	1.0580	0.5030	**0.5947**	0.9422	**0.5838**
	MixUp	0.6267	0.8571	0.6284	0.4375	1.1723	0.4419	0.5317	0.9664	0.5324
	RandAug	0.4386	1.1665	0.4179	0.5312	0.9977	0.4590	0.4370	0.9773	0.4176
	CutMix	0.5260	1.1346	0.5129	0.3750	1.0690	0.3604	0.4896	**0.9406**	0.4860
InceptionV3	AugMix	0.4191	1.3365	0.4171	0.3125	1.2287	0.2874	0.3844	1.0467	0.3771
	MixUp	0.6946	0.8153	0.6542	0.5625	0.9258	0.5160	0.4425	1.0506	0.4145
	**RandAug**	0.4676	1.3531	0.4532	0.4062	1.1455	0.4040	**0.5422**	**1.0297**	**0.5453**
	CutMix	0.4571	1.2502	0.4319	0.4375	1.1371	0.4307	0.5000	1.0810	0.4939
DenseNet121	AugMix	0.5025	1.1260	0.4964	0.4688	1.1029	0.3702	0.5000	1.0766	0.4053
	**MixUp**	0.6982	0.7466	0.6713	0.6250	0.7675	0.6056	**0.6210**	**0.7763**	**0.6220**
	RandAug	0.4066	1.3358	0.4048	0.3750	1.1619	0.3075	0.4841	1.0708	0.4172
	CutMix	0.4382	1.3478	0.4228	0.4375	0.9331	0.4035	0.4841	0.9894	0.4774
CNN	**AugMix**	0.5855	0.8824	0.5557	0.4375	1.0676	0.3454	**0.4524**	1.1120	**0.4241**
	MixUp	0.5909	0.9263	0.5576	0.3750	1.1183	0.1818	0.2684	1.3455	0.1410
	RandAug	0.3999	1.0676	0.3737	0.2188	1.3920	0.1197	0.4420	**1.0863**	0.2043
	CutMix	0.5893	0.9412	0.5405	0.2188	2.1327	0.1197	0.2842	1.2836	0.1475

**Table 9:** Performance of deep learning models on ALL-subtype multiclass classification (Benign, Early, Pre, Pro) across two datasets under four augmentation strategies (AugMix, MixUp, RandAug, and CutMix); trained for up to 100 epochs with early stopping.

Model	Augmentation	ALL (Benign, Early, Pre, Pro) (1)						ALL (Benign, Early, Pre, Pro) (2)					
		Validation			Test			Validation			Test		
		Acc.	Loss	F1	Acc.	Loss	F1	Acc.	Loss	F1	Acc.	Loss	F1
Xception	AugMix	0.9531	0.1286	0.9483	0.9295	0.1449	0.9082	0.9869	0.0389	0.9868	0.9839	**0.0410**	0.9838
	**MixUp**	0.9719	0.0898	0.9687	0.9815	**0.0845**	0.9756	0.9945	0.0402	0.9944	**0.9917**	0.0497	**0.9919**
	RandAug	0.9563	0.1567	0.9544	0.9485	0.1611	0.9437	0.9713	0.0854	0.9712	0.9655	0.0987	0.9647
	**CutMix**	0.9719	0.1503	0.9697	**0.9891**	0.1494	**0.9884**	0.9869	0.1515	0.9869	0.9850	0.1514	0.9852
VGG16	AugMix	0.9531	0.1220	0.9467	0.9611	0.1071	0.9505	0.9909	0.0348	0.9908	0.9928	0.0285	0.9928
	**MixUp**	0.9781	0.0729	0.9748	**0.9788**	**0.0700**	**0.9737**	1.0000	0.0224	1.0000	**0.9995**	**0.0222**	**0.9995**
	RandAug	0.9406	0.1617	0.9335	0.9659	0.1112	0.9615	0.9945	0.0234	0.9944	0.9924	0.0247	0.9925
	CutMix	0.9719	0.1758	0.9689	0.9770	0.1618	0.9732	0.9955	0.1324	0.9955	0.9938	0.1318	0.9938
ResNet50	AugMix	0.6969	0.8004	0.6715	0.7301	0.7485	0.6706	0.8286	0.4756	0.8287	0.8297	0.4786	0.8281
	**MixUp**	0.8594	0.4534	0.8543	**0.8555**	**0.4603**	**0.8448**	0.8831	0.3624	0.8830	**0.8870**	**0.3615**	**0.8856**
	RandAug	0.7000	0.8688	0.6122	0.6827	0.8680	0.5903	0.7888	0.6783	0.7889	0.7760	0.6681	0.7735
	CutMix	0.7656	0.6030	0.7280	0.8009	0.5254	0.7582	0.8760	0.4458	0.8757	0.8761	0.4411	0.8753
MobileNetV2	**AugMix**	0.9750	0.0658	0.9723	**0.9863**	**0.0587**	**0.9821**	0.9904	0.0283	0.9903	0.9965	**0.0200**	0.9965
	**MixUp**	0.9656	0.1126	0.9615	0.9743	0.0912	0.9679	0.9975	0.0271	0.9975	**0.9975**	0.0282	**0.9975**
	RandAug	0.9719	0.1126	0.9709	0.9812	0.1015	0.9815	0.9834	0.0590	0.9832	0.9808	0.0517	0.9807
	CutMix	0.9594	0.2006	0.9574	0.9651	0.1928	0.9582	0.9884	0.1326	0.9884	0.9871	0.1274	0.9869
InceptionV3	AugMix	0.9250	0.2000	0.9206	0.9273	0.2384	0.9152	0.9693	0.0791	0.9688	0.9656	0.0929	0.9649
	**MixUp**	0.9438	0.1892	0.9430	**0.9598**	**0.1454**	**0.9554**	0.9864	0.0607	0.9862	**0.9898**	**0.0588**	**0.9898**
	RandAug	0.8813	0.2649	0.8688	0.9512	0.1685	0.9474	0.9622	0.1122	0.9617	0.9581	0.1128	0.9581
	CutMix	0.9500	0.2257	0.9489	0.9388	0.2275	0.9364	0.9738	0.1627	0.9737	0.9726	0.1613	0.9724
DenseNet121	AugMix	0.9812	0.0691	0.9779	0.9794	0.0639	0.9745	0.9980	0.0106	0.9980	0.9961	**0.0144**	0.9962
	**MixUp**	1.0000	0.0419	1.0000	**1.0000**	**0.0372**	**1.0000**	1.0000	0.0157	1.0000	**0.9982**	0.0195	**0.9982**
	RandAug	0.9812	0.0506	0.9798	0.9835	0.0491	0.9819	0.9919	0.0274	0.9919	0.9940	0.0220	0.9940
	CutMix	0.9906	0.1336	0.9886	0.9881	0.1219	0.9862	0.9960	0.1089	0.9960	0.9946	0.1112	0.9946
CNN	**AugMix**	0.9750	0.1244	0.9717	**0.9842**	**0.1109**	**0.9801**	0.9904	0.0449	0.9904	0.9890	**0.0385**	0.9888
	**MixUp**	0.9719	0.1221	0.9668	0.9617	0.1282	0.9479	0.9955	0.0607	0.9954	**0.9942**	0.0502	**0.9943**
	RandAug	0.7563	0.7743	0.6189	0.8205	0.6452	0.6713	0.9693	0.1151	0.9692	0.9800	0.0828	0.9799
	CutMix	0.8625	0.4241	0.8457	0.8994	0.3721	0.8954	0.9763	0.1995	0.9761	0.9729	0.1687	0.9734

**Table 11:** Comparison of leukemia diagnostic models with XAI integration.

Model	Images	Dataset/Repository	Type of Medical Diagnostics	Method	XAI Technology Used	Accuracy
[2]	889	ALL-IDB	ALL, AML, CLL, CML	VGG, Xception, InceptionResV2, DenseNet, ResNet with RF and XGBoost	-	100% for ALL
		Private dataset (AML, CLL, CML)				97.08% for (AML, CLL, CML)
[7]	1250	AIIMS Patna	ALL, AML, CLL, CML	VGG16 with SVM	Grad-CAM	84%
[8]	889	ALL-IDB	ALL, AML, CLL, CML	VGG-16 and InceptionV3	LIME	83.33% (ALL)
		Private dataset (AML, CLL, CML)				100% (AML, CLL, CML)
Our model	20,000	As described in Section 4.1	ALL, AML, CLL, CML, H	MobileNetV2 with MixUp Augmentation	LIME, Grad-CAM	97.9%

## Data Availability

To allow reproducibility by other researchers and improvements on our work, the notebook codes for all models used in the study have been made available at: https://drive.google.com/drive/folders/1QnGBmsojl_zqUcE8rkYgH5vkEQjonnws?usp=sharing (accessed on 1 August 2024).
